# A Case of Late Radiation-Induced Enteritis with Enterolith Caused Enterocutaneous Fistula

**DOI:** 10.70352/scrj.cr.25-0197

**Published:** 2026-01-23

**Authors:** Kiyoe Takai, Tetsu Yamamoto, Takahito Taniura, Kazunari Ishitobi, Keisuke Inoue, Shunsuke Kaji, Takeshi Matsubara, Masaaki Hidaka

**Affiliations:** Department of Digestive and General Surgery, Shimane University Faculty of Medicine, Izumo, Shimane, Japan

**Keywords:** radiation-induced enteritis, enterolith, enterocutaneous fistula, radiation injury, short bowel syndrome

## Abstract

**INTRODUCTION:**

Radiotherapy for pelvic malignancies contributes to improved patient survival. However, early and late radiation-induced complications are increasing and cause significant impairment of quality of life. We reported a case of late radiation-induced enteritis associated with an enterocutaneous fistula suggestive of enterolith, which required 2 times of surgical treatments.

**CASE PRESENTATION:**

A 70-year-old female in a postoperative state of bladder cancer was treated with neoadjuvant chemotherapy followed by surgery. Total radiotherapy at 61.2 Gy was performed 1 month after surgery. The patient had intestinal obstruction 6 months after completing irradiation. As the intestinal obstruction was refractory to conservative treatment, she underwent surgical treatment. Because of the difficulty of adhesiolysis, bypass surgery between the jejunum at 150 cm from the Treitz ligament and the ascending colon was performed. The postoperative course was good. However, an enterocutaneous fistula, likely associated with an enterolith, occurred 45 months after surgery. The development of enterolith was considered to be due to intestinal stenosis associated with late-induced radiation injury. Because the conservative treatment did not improve, she had to undergo surgical treatment. The surgical findings showed that most of the small intestine was adhered and immobilized. An enterocutaneous fistula was formed through the abscess with an enterolith. A massive intestinal resection was performed, resulting in a short bowel syndrome. Macroscopically, edematous changes in the submucosal layer of the wall and thickening of the ileum were observed. Histopathological examination revealed fibrosis and vascular obstruction in the mesentery. Atrophy and reduced crypts of the villi, as well as eosinophil infiltration, were observed, indicating the occurrence of late radiation injury. Therefore, we diagnosed that enterolith due to radiation-induced late enteritis caused intestinal obstruction and enterocutaneous fistula. Three years after intestinal resection, there was no recurrence of bowel obstruction and no evidence of severe malnutritional status.

**CONCLUSIONS:**

Radiotherapy improves the survival of cancer patients. Nevertheless, irreversible and progressive late radiation-induced enteritis needs to be considered. Intensive follow-up is required to avoid severe complications or provide early treatment.

## Abbreviations

Alb
: albumin
CE-CT
: contrast-enhanced CT
CMV
: cytomegalovirus
CRP
: C-reactive protein
PNI
: prognostic nutritional index
RT
: radiotherapy

## INTRODUCTION

RT for pelvic malignancies is widely used. It contributes to improved patients’ survival, but it is well known that radiation-induced enteritis occurs frequently due to the inevitable irradiation of the intestine adjacent to the target organs.^1)^ Radiation-induced enteritis is roughly divided into early and late injury. Early injury typically occurs within weeks of irradiation, while late injury develops several months after irradiation. Early injury of RT is characterized by an inflammatory change in the intestinal epithelial cells resulting from direct irradiation damage, which is typically transient and resolves with conservative treatment. On the other hand, late injury of irradiation can lead to serious complications such as stenosis and perforation, which require surgical treatment.

Primary enterolith is a rare condition, classified into 2 subcategories: “true” and “false”.^2)^ True enteroliths mainly consist of normal intestinal fluid, whereas false enteroliths are formed from ingested indigestible substances. True enteroliths caused by irradiation are extremely rare.^3)^ Moreover, nontraumatic perforation of the small intestine is an infrequent event.^4)^

We reported a case of late radiation-induced enteritis after radical RT for bladder cancer, which required 2 surgical treatments due to stenosis and perforation suggestive of enterolith.

## CASE PRESENTATION

**Fig. 1:**
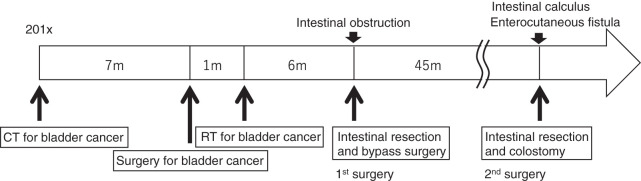
Patient’s treatment timeline.

**Fig. 2:**
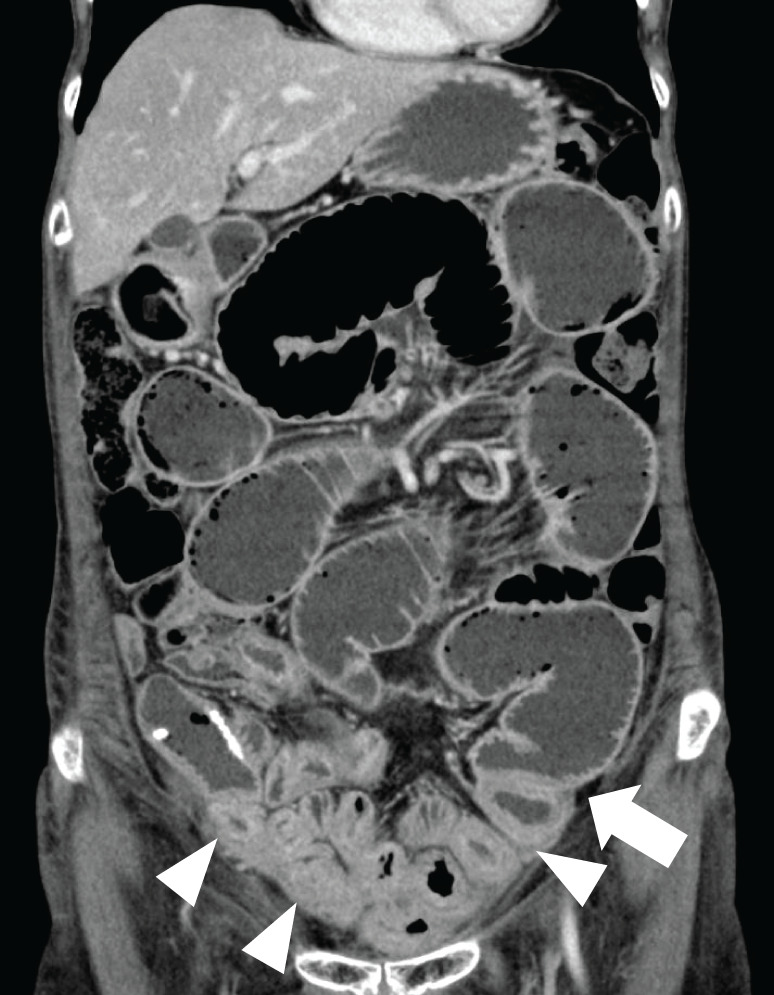
Coronal view of abdominal CE-CT at the initial visit represents wall thickening (arrowhead) and caliber change (arrow) of the small intestine. CE-CT, contrast-enhanced CT

A 70-year-old female presented with lower abdominal pain. She had undergone treatment for bladder cancer (pT4bN0M0 pStage IV, 7th edition of the UICC/AJCC TNM), 4 cycles of gemcitabine plus cisplatin therapy starting from February 2010, and total cystectomy with ileal conduit surgery in September 2010. RT (50.4 Gy/28 fr for the small pelvis and 10.8 Gy/6 fr for the tumor bed, a total of 61.2 Gy) was performed 1 month after surgery (**Supplementary Fig. 1**). The normal uterus existed with an age-appropriate size. Furthermore, the irradiation field was sufficiently distant from the ileum, ileocecal valve, and Treitz ligament. No adverse event was observed during the irradiation period. However, intestinal obstruction occurred 6 months later. Because conservative treatment did not show improvement, she had to undergo surgical treatment (**Fig. 1**). Significant thinning was observed in height (150 cm), weight (37.9 kg), and BMI (16.8 kg/m^2^). The abdomen was distended, soft, and tender. A median incisional scar at the lower abdomen and a urostomy with ileal conduit in the right lower abdomen were observed. The laboratory data revealed only anemia with a hemoglobin of 9.9 g/dL (reference range 11.6–14.8). For nutritional assessment, Alb was 3.1 g/dL (4.1–5.1), and PNI was 34.0, indicating a low nutritional status. A mildly elevated inflammatory response was observed, with a CRP level of 3.65 mg/dL (≤0.14). Imaging examinations showed that abdominal and pelvic CE-CT scans at the time of initial consultation showed wall thickening of the small intestine in the pelvis and increased density of the fat tissue surrounding the mesentery. Caliber change and intestinal dilatation at the oral side were observed (**Fig. 2**). The final diagnosis was intestinal obstruction due to late radiation-induced enteritis, and the patient was scheduled to undergo an elective adhesiolysis with intestinal resection.

### Initial surgery

She was placed in the supine position under epidural and general anesthesia. The abdomen was incised along with the surgical scar in the lower abdomen. The ileum was located in the pelvis and was distorted and immobile due to severe adhesions, also known as frozen pelvis following radiation therapy. The oral intestine was markedly dilated. Because of the difficulty of adhesiolysis, bypass surgery between the jejunum at 150 cm from the Treitz ligament and the ascending colon was performed.

The postoperative course was good, and a 1-year outpatient follow-up was completed. However, left lower abdominal pain and stool discharge from the midline scar of the lower abdomen appeared 45 months after surgery.

**Fig. 3:**
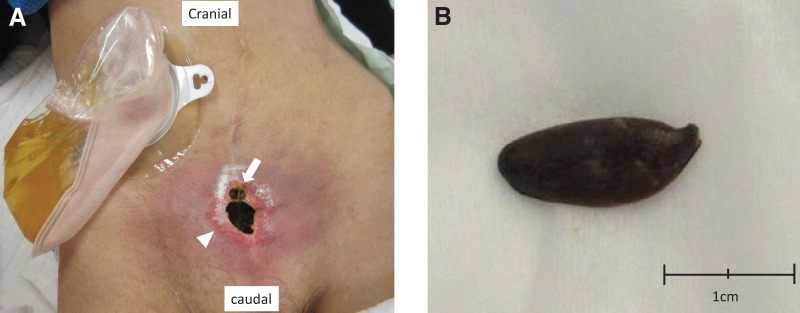
Abdominal findings before the second surgery. (**A**) The enterocutaneous fistula was observed on the operation scar above the 3-cm pubic tubercle (arrow head). Stool discharge and enterolith were observed (arrow). Ileal conduit was located approximately 15 cm to the right and superior to the enterocutaneous fistula. (**B**) About 1.5 × 0.8 cm of enterolith was removed from the enterocutaneous fistula.

**Fig. 4:**
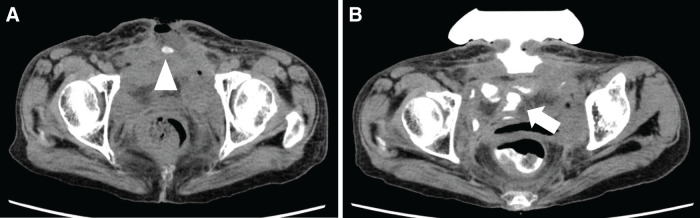
(**A**) Plain CT imaging showed an intra-abdominal abscess and a high concentration of stone just below the open wound (arrowhead). (**B**) Fistulography showed an influx of contrast agents into the intestinal tract (arrow).

An open wound about 3 cm in the midline scar of the lower abdomen and internal juice discharge and enterolith were observed (**Fig. 3A** and **3B**). CT scan showed an abscess cavity and a high concentration of stone under the open wound (**Fig. 4A**). Influx of contrast into the bowel was recognized by fistulography using urographin (**Fig. 4B**).

### Second surgery

**Fig. 5:**
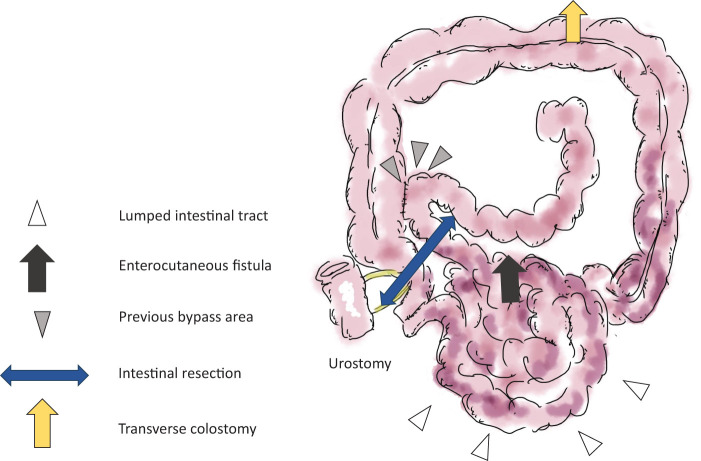
Schema of the second surgery showed that the anal side of the intestinal tract from the previous bypass surgery site (gray arrowhead) was lumped together in the pelvic cavity (white arrowhead), and an enterocutaneous fistula was formed through the abscess cavity (black arrow). The lumped intestine was resected (blue double arrow). A transverse loop colostomy was made (yellow arrow).

**Fig. 6:**
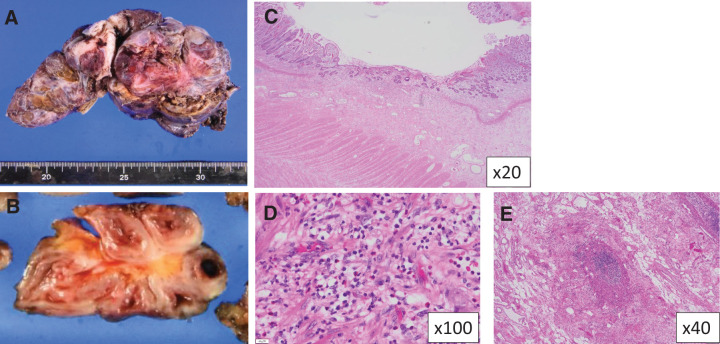
(**A**) About 15 cm of mass formation with radiation-injured intestine was resected at a second surgery. (**B**) The edematous mucosal layer and wall thickening of the seromuscular layer were observed. (**C**) HE staining showed atrophy of the villi and reduction of the crypts (HE ×20). (**D**) Eosinophil infiltration was observed in the mucosal and submucosal layers (HE ×100). (**E**) Blood vessel obstruction was observed in the subserosal layer and the intramesentery (HE ×40).

The remnant small intestine in the pelvis at the time of the first surgery was still distorted and immobilized, and an enterocutaneous fistula was formed through the abscess. Due to severe adhesions likely associated with radiation-induced late injury around the descending colon and rectum, adhesiolysis was difficult. Therefore, the previous bypass area was preserved, and the perforated small intestine, including the abscess and enterocutaneous fistula, was resected. A transverse loop colostomy was made because of the high risk of future stenosis of the rectum. The length of the residual small intestine was approximately 150 cm (**Fig. 5**). Macroscopically, edematous changes in the submucosal layer and wall thickening of the ileum were observed. Histopathological examination revealed fibrosis and vascular obstruction in the mesentery. Atrophy and reduced crypts of the villi, as well as eosinophil infiltration, were observed. The increasing fibroblast and myofibroblast proliferation were observed on the subserosal layer. Occlusion of blood vessels due to infiltration of inflammatory cells, such as histiocytes, was observed (**Fig. 6A**–**6E**). These findings indicate that late injury after radiation therapy was presented in the resected small intestine.

Although grade IIIa SSI occurred around the resected fistula area, the patient’s postoperative course was almost good, and the patient was discharged on the 38th POD. Short bowel syndrome was a concern, but she was in good nutritional status, with a BMI of 20.4, Alb of 4.0 g/dL, and a PNI of 47.1, achieved solely through oral intake, at 3 years postoperatively.

## DISCUSSION

Radiation-induced enteritis is a disorder of the intestinal tract secondary to abdominal and pelvic irradiation and was reported by Walsh in 1897.^5)^ The frequency is reported to be approximately 5%–55%, which varies depending on the irradiation method, dose, and radiosensitivity of the organs.^6,7)^ Radiation-induced enteritis is reported to be particularly susceptible in the elderly and in patients with systemic diseases, such as lupus, hypertension, diabetes, collagen disease, and renal insufficiency.^8)^

Radiation-induced enteritis is classified according to the time of onset, with early injury, which develops during or within 3 months of radiation therapy, and late injury, which appears more than 3 months after the end of irradiation.^9)^ The mechanisms differ between the early and late stages of injury. The early injury caused by direct damage to the intestinal epithelial cells through irradiation results in mucosal edema and impaired blood flow.^10)^ The symptoms of early intestinal toxicity (nausea, abdominal pain, diarrhea, and fatigue) occur in 60%–80% of patients during radiation therapy.^9)^ These changes and symptoms are transient and reversible as long as the cellular regenerative capacity is caught up.

On the other hand, late injury is thought to be caused by a decrease in the wound-healing process due to radiation-induced inflammation, including impaired tissue regeneration and delayed wound healing, resulting from a decrease in residual stem cells. Persistent vascular permeability leads to fibroblast deposition and, ultimately, to the thickening of the vascular intima.^10,11)^ In general, fibrosis develops and intensifies over 6 months after irradiation, and gradual vascular occlusion leads to scarring and hyalinization of the tissue.^12)^ This scarring and hyalinization are irreversible and progressive, and they may lead to intestinal obstruction in severe cases. It also leads to stricture, fistula formation, perforation, and massive bleeding, which usually needs surgical treatment.^1)^ In a Phase III randomized controlled trial of postoperative radiotherapy for cystitis, late complications were reported to occur in 18%–27% of cases.^13)^ On the other hand, for late gastrointestinal complications, Grade 2 or higher complications reports indicate Grade 2 or higher complications in approximately 35% of cases.^14)^ However, no reports of enterocutaneous fistulae were identified.

In this case, severe adhesions were observed during surgery, and characteristic pathological findings were also confirmed, suggesting that the obstruction resulted from late radiation injury. Differential diagnoses, such as Crohn’s disease, NSAID enteropathy, ischemic enteritis, infections (tuberculosis, CMV), and malignant recurrence, were excluded by CT findings, pathology, and tumor markers. However, microbiological examination was not performed.

There are no reports on the incidence of enterocutaneous fistula formation following radiation therapy to the pelvic region. However, regarding intestinal fistulas (including enterocutaneous fistula), a meta-analysis reported that the incidence rate for radiation therapy for prostate cancer was 0.2% across all 6 cohort studies.^15)^ However, the incidence rate varies depending on the type of cancer and the treatment method (chemotherapy or radiation dose). Additionally, a PubMed search using the 3 keywords “enterolith”, “enterocutaneous fistula,” and “radiation enteritis” yielded no relevant literature, indicating that the simultaneous occurrence of these 3 conditions is extremely rare. In this case, enterolith formation was observed after bypass surgery. Whether the calculus in this case was a true or false enterolith remains unclear, because the analysis of enterolith composition was not performed. However, we believe this enterolith could have been true. The reason for this is as follows: Although true enteroliths are rare, intestinal stasis was observed in this case, suggesting that calculi could have formed even with normal intestinal fluid. While preoperative malnutrition contributes to delayed healing and leakage, multiple factors likely play a role after radiotherapy. Therefore, we believe the enterolith was the direct cause of fistula formation. This is supported by several reports describing enteroliths associated with radiation-induced enteritis.^16,17)^

Surgical treatment includes bowel resection and reconstruction, bypass, and colostomy. Although bowel resection and reconstruction are most effective in improving abdominal symptoms, it is often difficult to perform in cases of severe adhesions, known as frozen pelvis.^7)^ These patients have a high risk of delayed wound healing and anastomotic leakage, with contributing factors potentially including not only nutritional status but also the effects of radiation therapy and other clinical factors. It is also suggested that massive resection of the small intestine may cause a short bowel syndrome, which significantly reduces the quality of life.^18)^ Bypass surgery has the advantage of being simple in technique and avoiding short bowel syndrome. However, there is a possibility that a blind loop or malabsorption syndrome, caused by dysfunction of the remaining intestine, may necessitate reoperation.^19)^ Radiation-induced enteritis is irreversible and progressive; therefore, long-term follow-up should be considered when performing bypass surgery. The reason is that chronic stasis of intestinal contents, secondary to atrophy and dysfunction of the remaining intestinal mucosa, may lead to the formation of enteroliths. The enteroliths cause mucosal irritation and intestinal congestion, eventually leading to perforation and the formation of an enterocutaneous fistula.

Regarding the mechanism of radiation-induced enteritis, Takemura et al. reported that the interaction between eosinophils and myofibroblasts plays a crucial role in inducing fibrosis in in vivo studies. In addition, administration of an eosinophil-eliminating antibody markedly suppressed fibrosis after irradiation, suggesting that eosinophil targeting treatment may be a candidate for preventing radiation-induced enteritis as a non-surgical treatment.^20,21)^ The histopathology of this case revealed eosinophil infiltration in addition to fibrosis, suggesting that radiation-induced eosinophil infiltration may have played a significant role in the development of radiation-induced enteritis. Investigation of risk factors for radiation-induced enteritis and the development of new therapeutic agents, such as antibodies for high-risk cases, are urgently needed.

### Limitation

The consistency of the enterolith is an estimate, since we did not examine its composition. The initial bypass procedure could have contributed to long-term stasis and enterolith formation. However, since such a quantitative evaluation is impossible, it remains merely a possibility.

## CONCLUSIONS

We reported a case of late radiation-induced enteritis, which was suggestive of a complicated enterocutaneous fistula due to enteroliths. Although radiotherapy contributes to improved survival of cancer patients, irreversible and progressive late radiation-induced enteritis needs to be considered. Thus, intensive follow-up after radiotherapy is needed to avoid severe complications or provide early treatment.

## SUPPLEMENTARY MATERIALS

Supplementary Fig. 1 Treatment planning images and isodose maps for small pelvic region (**A** and **B**) and bladder floor (**C** and **D**). The dose of irradiation was 50.4 Gy for pelvic region and 10 Gy for bladder floor. The radiation field was distant from the ileal conduit.

## DECLARATIONS

### Availability of data and materials

The data that support the findings of this study are available from the corresponding author upon reasonable request.
