# Supplementary figures and images for: A Case of Late Radiation-Induced Enteritis with Enterolith Caused Enterocutaneous Fistula
Source: Surg Case Rep. 2026 Jan 23;12(1):25-0197. doi: 10.70352/scrj.cr.25-0197 (PMC12852806; doi:10.70352/scrj.cr.25-0197)

# Supplementary figure 1

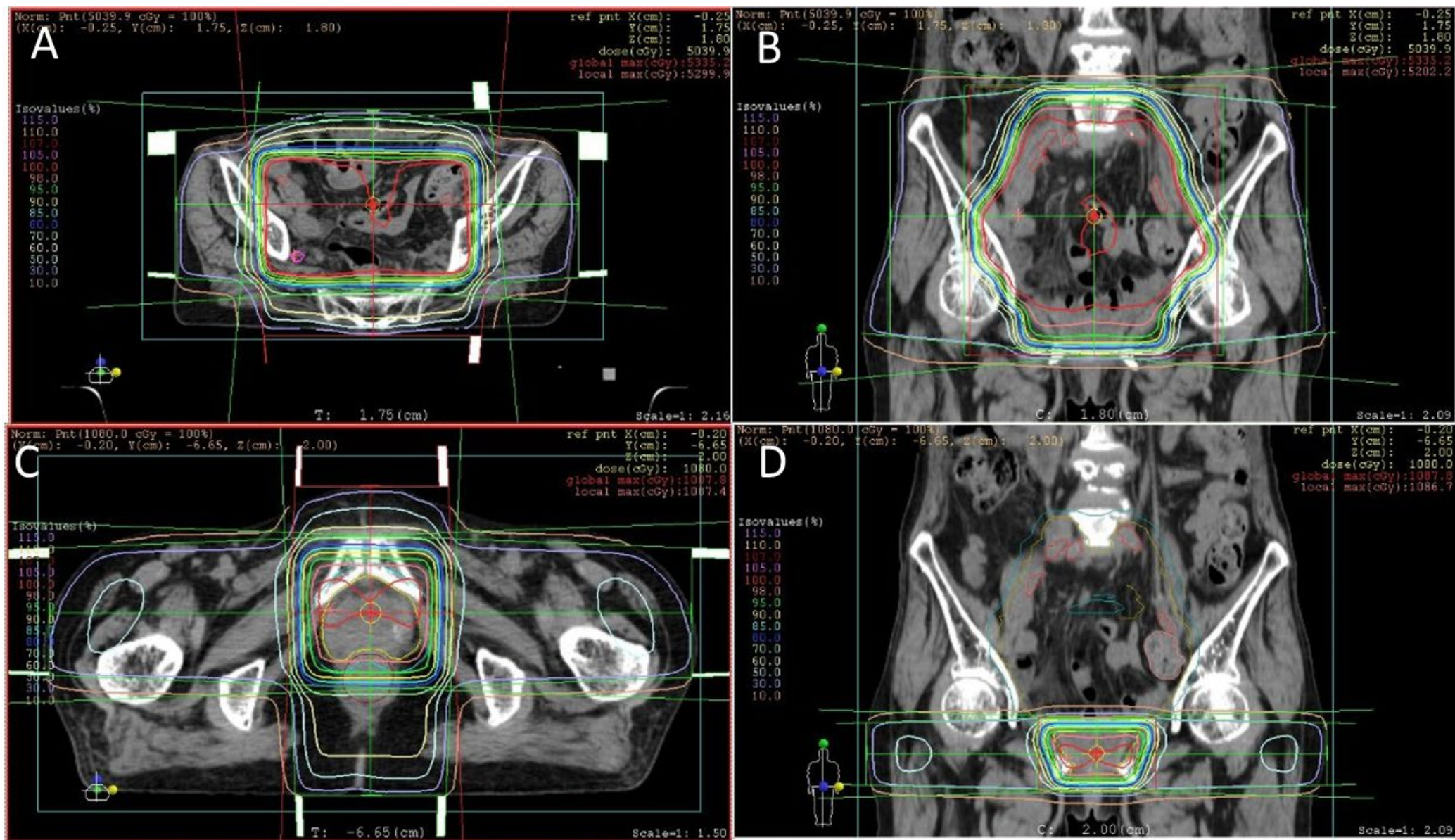

Supplement: Supplementary Fig. 1 — Treatment planning images and isodose maps for small pelvic region (A and B) and bladder floor (C and D). The dose of irradiation was 50.4 Gy for pelvic region and 10 Gy for bladder floor. The radiation field was distant from the ileal conduit. [file scr-12-01-25-0197-s001.pdf]
